# Cardiac myosin super relaxation (SRX): a perspective on fundamental biology, human disease and therapeutics

**DOI:** 10.1242/bio.057646

**Published:** 2021-02-15

**Authors:** Manuel Schmid, Christopher N. Toepfer

**Affiliations:** 1 Division of Cardiovascular Medicine, Radcliffe Department of Medicine, University of Oxford, Oxford OX3 9DU, UK; 2 Department of Genetics, Harvard Medical School, Boston, MA 02115, USA; 3 Wellcome Centre for Human Genetics, University of Oxford, Oxford OX3 7BN, UK

**Keywords:** Myosin, Super relaxed state (SRX), Interacting heads motif (IHM), Myosin mesa, Hypertrophic cardiomyopathy, Targeted therapy

## Abstract

The fundamental basis of muscle contraction ‘the sliding filament model’ (Huxley and Niedergerke, 1954; Huxley and Hanson, 1954) and the ‘swinging, tilting crossbridge-sliding filament mechanism’ (Huxley, 1969; Huxley and Brown, 1967) nucleated a field of research that has unearthed the complex and fascinating role of myosin structure in the regulation of contraction. A recently discovered energy conserving state of myosin termed the super relaxed state (SRX) has been observed in filamentous myosins and is central to modulating force production and energy use within the sarcomere. Modulation of myosin function through SRX is a rapidly developing theme in therapeutic development for both cardiovascular disease and infectious disease. Some 70 years after the first discoveries concerning muscular function, modulation of myosin SRX may bring the first myosin targeted small molecule to the clinic, for treating hypertrophic cardiomyopathy (Olivotto et al., 2020). An often monogenic disease HCM afflicts 1 in 500 individuals, and can cause heart failure and sudden cardiac death. Even as we near therapeutic translation, there remain many questions about the governance of muscle function in human health and disease. With this review, we provide a broad overview of contemporary understanding of myosin SRX, and explore the complexities of targeting this myosin state in human disease.

This article has an associated Future Leaders to Watch interview with the authors of the paper.

## Abstract (precis)

**Summary:** A discussion of the role and regulation of myosin super relaxation in biology, evolution, human disease, and cardiac therapeutics.

## Introduction

Fundamentally, myosins are able to produce force when they are available to bind actin and hydrolyse ATP. These interactions drive the sliding of thick and thin filaments past one another, contracting sarcomeres, and creating the force and muscular shortening that allows the heart to circulate blood. If myosins are not in close proximity to actin they cannot use ATP to generate force and drive muscular contraction. Filamentous myosins that are available to bind actin exist in a wide range of structural states and proximities to the thin filament, these conformations have more recently been defined as the disordered relaxed state (DRX) (Cooke, 2011; Fusi et al., 2015; Stewart et al., 2010). When viewed by electron microscopy or indirectly by X-ray diffraction, these active states occupy a wide variety of structures and proximities to the thin filament, and look ‘disordered’ (Cooke et al., 1982; Thomas and Cooke, 1980). DRX myosin is in balance with a well-ordered state of myosin termed the super relaxed state (SRX). Myosin SRX is a biochemical and likely structural state, where myosin heads interact with one another and the thick filament backbone (Alamo et al., 2016; Hooijman et al., 2011). These interactions form the basis of structural sequestration of myosin heads away from the thin filament, typified by deposition of these catalytic domains onto the thick filament backbone, providing the more ordered structure, myosin SRX (Alamo et al., 2008; Huxley and Brown, 1967; Woodhead et al., 2005). SRX myosin heads are unavailable for binding actin to produce force. This is a curious characteristic of myosin regulation within a filament; why would a cell produce a protein for it to be dormant? What is becoming clearer is that myosin SRX is one of the key determinants of the constant N_a_ (cross bridge number) in Huxley's model of force production, and that it is incredibly important to regulate the activity of these motors to ensure mechanical and energetic homeostasis within the cell. Especially pertinent in cardiac myocytes, which do not readily divide, cannot be replaced, and must contract and relax on average 60 times a minute throughout an individual's entire life.

Myosin SRX was first defined with its discovery in rabbit skeletal muscle (Stewart et al., 2010; Myburgh et al., 1995). SRX was found to account for a significant proportion of myosin in cardiac thick filaments (Hooijman et al., 2011). Rather than switching the motor unit ‘on’ and ‘off’ to allow contraction like in smooth muscle myosin (Cross et al., 1988), myosin SRX in cardiac muscle is more modulatory and provides more graded recruitment of myosin heads and force production (Hooijman et al., 2011). This difference goes hand in hand with the functional demands of both cardiac and skeletal muscle. Skeletal muscle remains in a relaxed state as long as no force is needed, and is able to rapidly adapt cross-bridge recruitment to satisfy demand. In contrast, cardiac muscle must contract and relax on average 60 beats a minute over the lifetime of an individual, while contracting in a graded and tightly moderated manner, which is adapted gradually not beat to beat, to raise or lower cardiac output (Kobirumaki-Shimozawa et al., 2014; McNamara et al., 2015). There are many properties of the sarcomere that influence recruitment of myosin cross-bridges from the SRX state. These include pre-load of the ventricle and post-translational modification. It has become clear that SRX recruitment contributes to the Frank-Starling law and is important in adapting cardiac output (Marcucci et al., 2019; Reconditi et al., 2017). Myosin SRX has its fundamental roots in earlier evolutionary phylogenies of myosins, and is intricately controlled by the interactions that form the basis of the myosin interacting heads motif (IHM) (Alamo et al., 2016). Myosin SRX dysregulation has been observed as a driving mechanism in HCM. Modulation of myosin SRX has become a central theme for the first targeted therapeutics for genetic cardiomyopathies (Repetti et al., 2019b). The utility of targeting myosin SRX therapeutically may extend beyond inherited cardiac conditions to acquired cardiovascular disease and even infectious disease (Trivedi et al., 2020).

## The evolutionary origin of the myosin interacting heads motif (IHM) and the myosin super relaxed state (SRX)

**Fig. 1.**
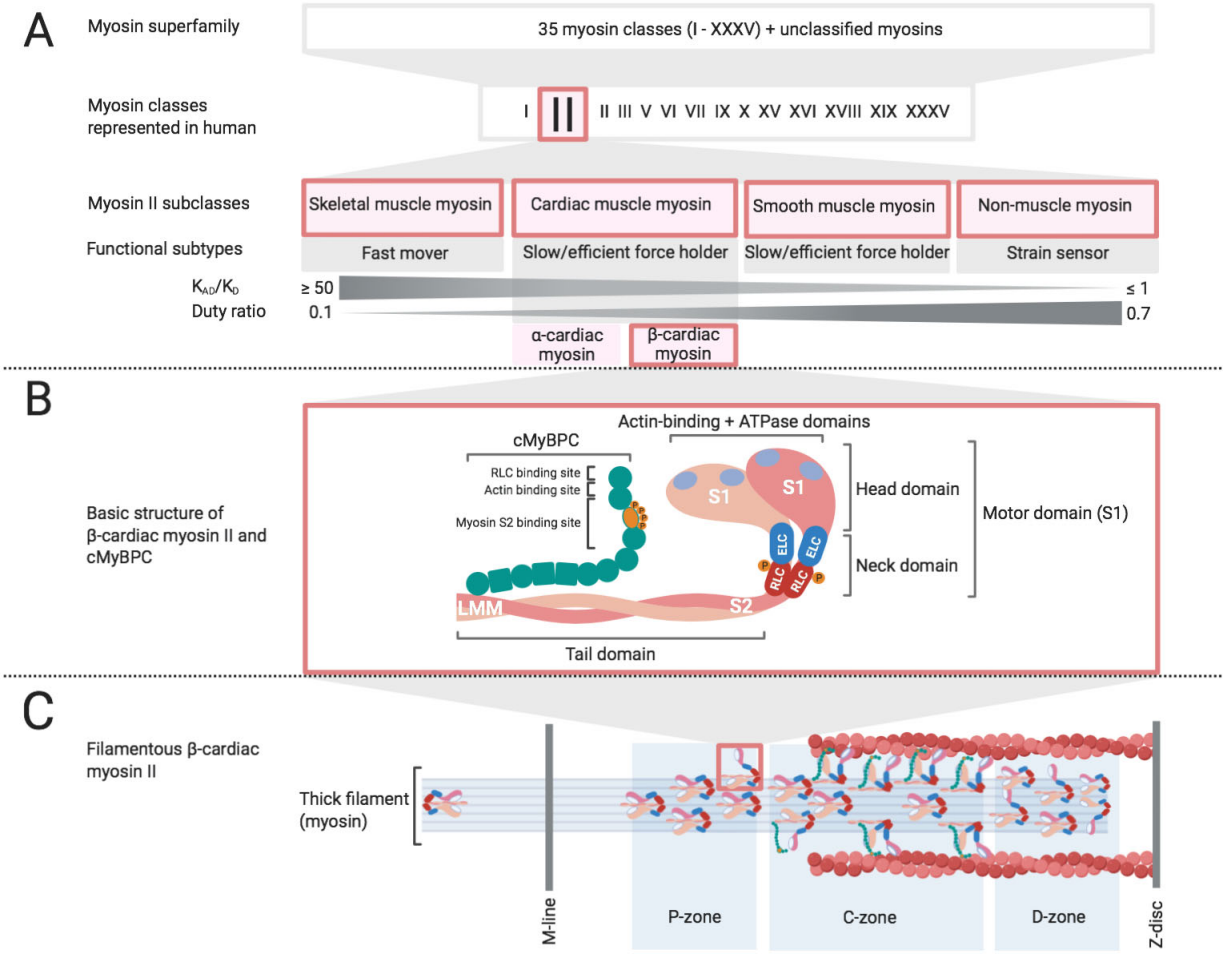
**Classification, functional and structural hallmarks of Myosin II.** (A) 35 groups of myosin (I-XXXV) have been classified based on the phylogenetic relation of their motor domains where unclassifiable myosins are denoted as orphan myosins (Odronitz and Kollmar, 2007). Subclasses of Myosin II include striated, smooth and non-muscle myosin, functionally differing from one another by duty ratio and thermodynamic coupling (Bloemink and Geeves, 2011). β-cardiac myosin II is primarily expressed in myocardium and encoded by the gene MYH7. (B) β-cardiac myosin II is formed by the characteristic two-headed structure with motor domains, neck regions including ELC and RLC, and a coiled-coiled tail domain. **Inset** cMyBPC exhibits integral binding sites for sarcomeric proteins. (C) Illustration of a half-sarcomere, which shows zone-specific myosin head conformations adapted from Brunello et al., 2020. The majority of actin-attached and force producing myosin heads are located in the C-zone whereas proximal (P) and distal (D) zones primarily comprises disordered and folded myosin heads.

Myosin is an actin-based motor protein that hydrolyzes ATP driving the motile properties of cells, tissues, and organs. A wide variety of myosin motors exist in nature and they share an evolutionary ancestor with kinesins, the microtubule-based molecular motor. The diverse set of myosin molecular motors have been classified into subgroups by several criteria (Fig. 1A) (Bloemink and Geeves, 2011; Odronitz and Kollmar, 2007). All myosins have an amino-terminal catalytic head domain, which contain actin- and ATP-binding sites. The major structural differences are found in the carboxyl-terminal, also known as the tail region, which varies widely in length and sequence, and allows the diverse cellular functions that myosins play within cells (Sellers, 2000). Among all these subgroups, Myosin IIs, also referred to as conventional myosins, were the first to be discovered (Kühne, 1864), and the only myosin subgroup that is known to assemble filaments (Sellers, 2000).

Myosin II molecules are made up of two identical heavy chains, each of these possess a globular head, a coiled-coiled tail domain, and a neck region that links the head and tail (Fig. 1B). Each neck domain of a heavy chain possess two IQ motifs that act as binding sites for two light chains, known as the essential (ELC) and the regulatory light chain (RLC) (Bähler and Rhoads, 2002; Heissler and Manstein, 2013). The Myosin II phylogeny possess a wide range of functional subtypes, which differ from one another by their respective duty ratio and thermodynamic coupling (Fig. 1A). The duty ratio defines the fraction of the ATPase cycle that the myosin spends in a strongly bound state to actin, while thermodynamic coupling (K_AD_/K_D_) describes the ratio of ADP affinity for actomyosin, versus the ADP affinity for myosin (Bloemink and Geeves, 2011). This sub-classification highlights the functional diversity of Myosin IIs, which encompass non-muscle myosins that are integral to cytokinesis (Pollard, 2020) as well as cell adhesion, cell migration and tissue architecture (Vicente-Manzanares et al., 2009), smooth muscle myosins that line many of the visceral organs and the circulatory system, and skeletal and cardiac muscle myosins that are responsible for providing force and power to move and pump blood in the heart.

**Fig. 2.**
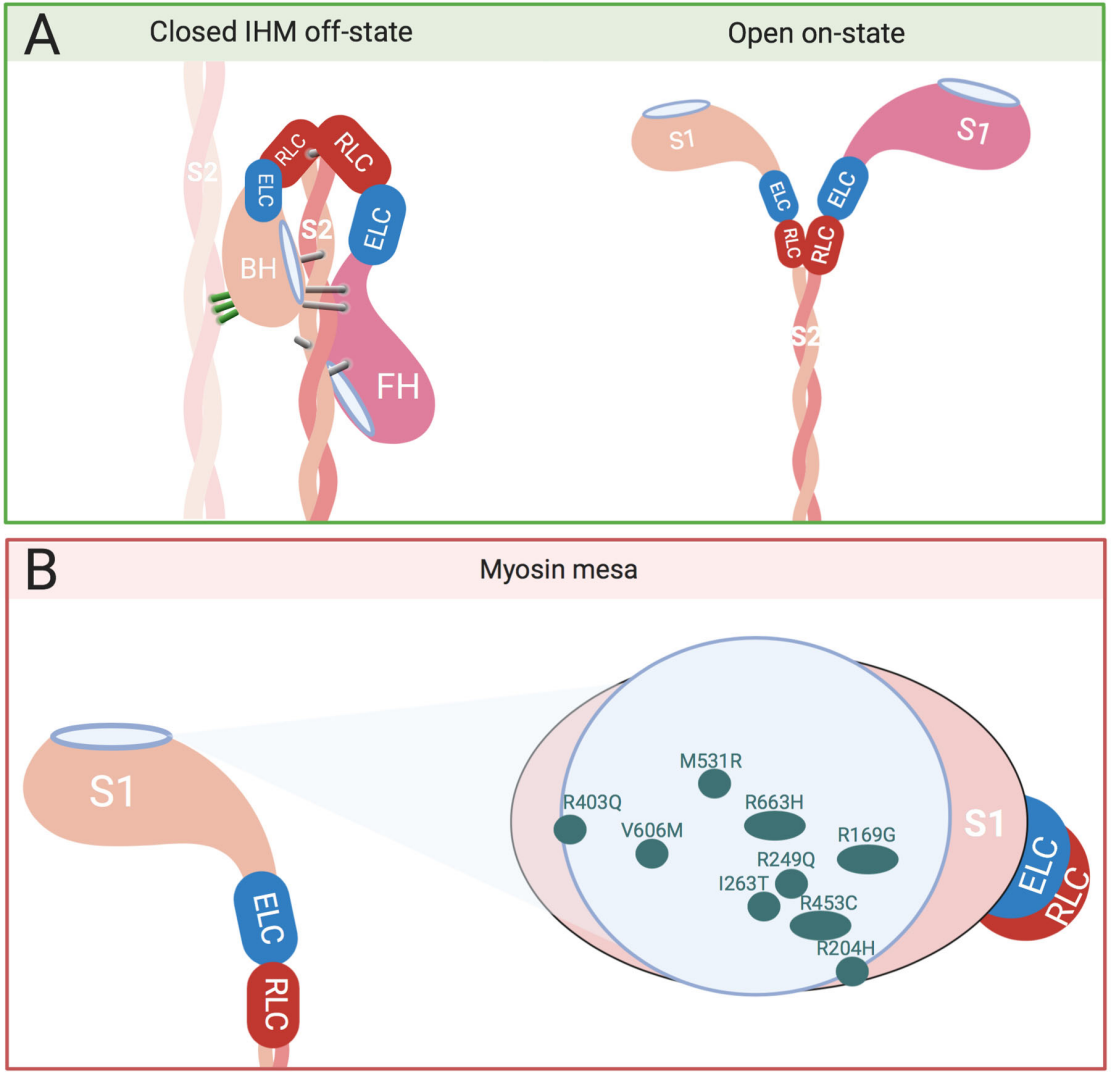
**The interacting Heads Motif (IHM) and the Myosin Mesa.** (A) The IHM interactions include the folded back conformation of the myosin dimer for interactions with the core of the thick filament, as well as interactions with adjacent myosin molecules (left) (adapted from Anderson et al., 2018). This is in contrast to the open on-state (right). Characteristic intermolecular (green) and intramolecular (grey) interactions defined by Alamo et al. maintain the IHM off structure, thus the blocked head (BH) is prevented from binding to actin by interacting with the free head (FH) (Alamo et al., 2016). The ATPase activity of the free head is inhibited through the attachment with the blocked head. (B) The side view (left) of the flat surface of a myosin head (left) shows the location of the myosin mesa. The top view (right) illustrates its relevance within the catalytic domain as a crucial location for well described HCM causing mutations displayed as green spots (adapted from Spudich, 2015).

What is clear is that all four subtypes of Myosin II have developed regulatory mechanisms to adapt energy consumption, to allow muscle relaxation, or to reduce myosin activity in cells not undergoing cellular division. This is a very important quality of these motors as molecular and cellular motion is energetically costly and needs to be tightly controlled and in balance with energy production within the cell. Even though smooth and nonmuscle Myosin IIs are distantly related to skeletal and cardiac muscle within the Myosin II phylogeny (Golomb et al., 2004), they all possess an IHM (Fig. 2A), a structure that is common to the Myosin II phylogeny. The Myosin IHM is highly conserved across the Myosin II phylogeny (González-Solá et al., 2014; Pinto et al., 2012; Woodhead et al., 2013; Zhao et al., 2009). One would predict that the IHM provided an evolutionary advantage for it to be so highly conserved. In both *Cnidaria* (the most primitive animals with muscles) (Sulbarán et al., 2015) and *Porifera* (considered to be the oldest animal phylum which lack muscle tissue) the IHM is present (Lee et al., 2018a,b). This suggests that the IHM motif emerged up to 700 and 900 million years ago (Lee et al., 2018a,b). Even in the eukaryote *Dictyostelium* modified folding of the Myosin tail similar to what one expects from the IHM has been found (Lee et al., 2018a,b). The formation of myosin molecules by the coiled-coiled domains of two myosin heavy chains brings myosin heads into close proximity at their amino terminus, this forms the basis of the IHM (Alamo et al., 2018). This folded structure of myosin has recently been resolved in spectacular detail in smooth muscle myosin by cryo-EM (Scarff et al., 2020; Yang et al., 2020). The IHM has been conserved and diversified evolutionarily, yet the IHMs importance in regulating cardiac muscle has largely been overlooked, until recently, when it became clear that residues within the IHM defined a significant proportion of cases of the genetic cardiomyopathy HCM (Alamo et al., 2017). SRX may have been adaptive even in earlier mammalian evolution during hibernation and in response to cold (Toepfer et al., 2020). States of myosin that are not fully described as either SRX or DRX have been observed by Caremani et.al. where mouse skeletal muscle cells at sub physiologic temperatures form a refractory state of myosin, that is not consistent with the sequestered structure of SRX nor the high nucleotide utilisation and cross-bridge formation of DRX (Caremani et al., 2019), a similarity with another well-ordered state observed when infusing muscle with 2′-deoxy-ATP (dATP) (Ma et al., 2020). These findings show that there is still much to be understood about the fundamental myosin states in relaxed and active muscle. But it seems that myosin SRX is an evolutionarily conserved energy saving mechanism that enables animals to have high-energy efficiency of muscular function, as an adaptation to survive and move in extreme conditions, such as during hibernation (Toepfer et al., 2020), or in the conditions that the wildebeest experiences during periods of nutritional deprivation and high ambient temperature (Curtin et al., 2018). SRX could also be essential in decreasing the metabolic rate of cardiomyocytes during periods of myocardial stress. This is an area of particular interest as myosin SRX regulation has not been studied in a variety of inherited and acquired cardiovascular diseases, including myocardial infarction, atrial fibrillation, heart failure with reduced ejection fraction (HFrEF), heart failure with preserved ejection fraction (HFpEF), amongst other cardiac conditions. One could hypothesise that during myocardial stunning/hibernation post-ischemia that myosin SRX could form as a consequence or in compensation to reduced blood flow. Indeed, if stunned myocardium does not form SRX one could therapeutically increase myosin SRX acutely to reduce metabolic demand during ischemia.

## The structure of myosin SRX and its relation to the IHM in cardiac muscle

**Fig. 3.**
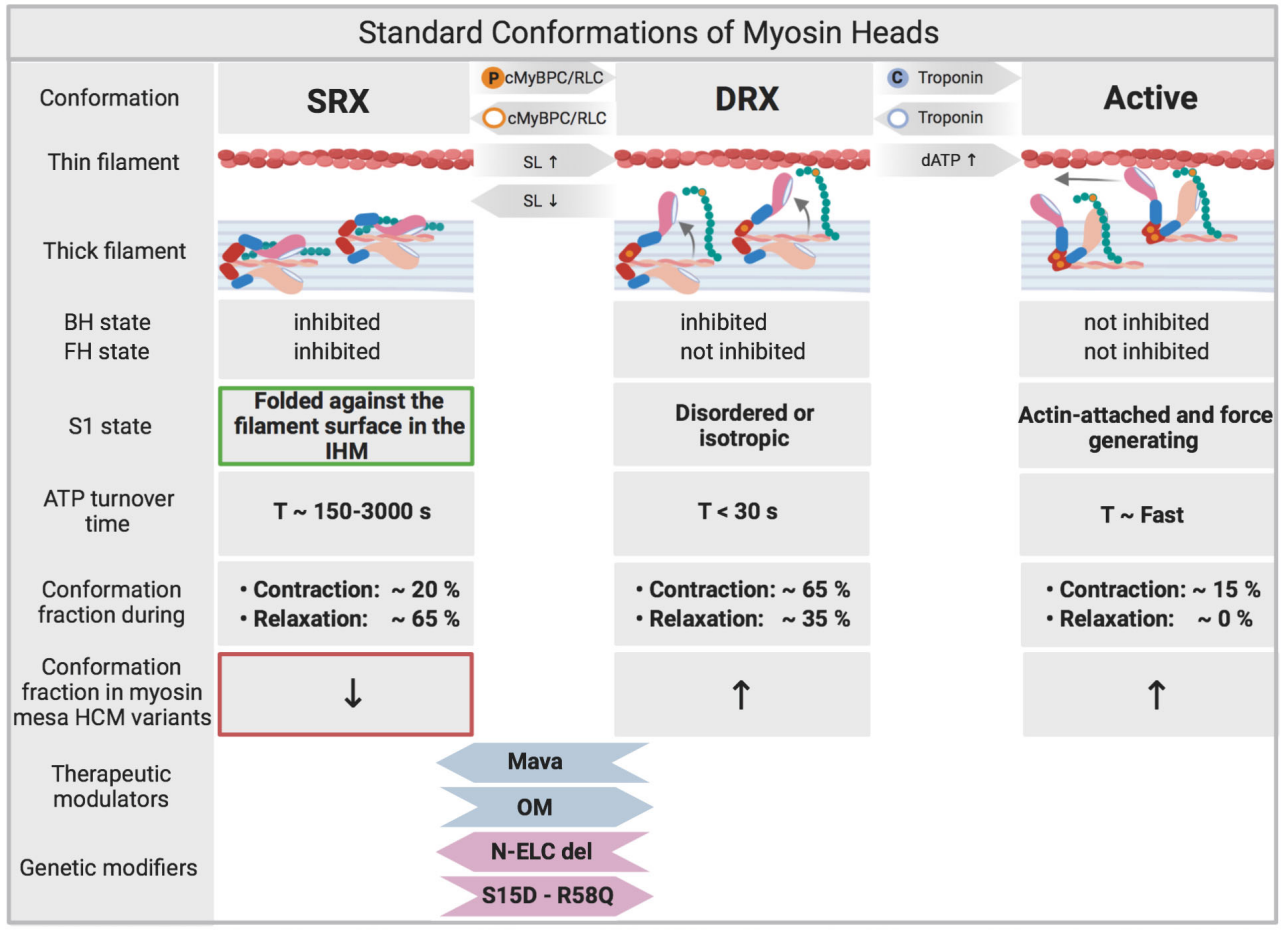
**Standard conformations of myosin heads.** Physiologically the SRX state and DRX state of myosin is balanced and subject to variation depending on regulating mechanisms such as cRLC and cMyBPC phosphorylation as well as sarcomere length (SL). Calcium binding to Troponin allows myosin heads to bind actin. SRX/DRX/Active states are dynamic over the time course of contraction. The majority of myosin heads remain in the SRX conformation during relaxation (Brunello et al., 2020). Reduced myosin SRX occurs in certain HCM causing mutations including those within the myosin mesa (Fig. 2B). Mavacamten (Alamo et al., 2016) has been shown to increase myosin SRX conformations (Rohde et al., 2018) (Anderson et al., 2018; Green et al., 2016; Olivotto et al., 2020). In contrast, Omecamtiv Mecarbil (OM) activates cardiac muscle contraction (Cleland et al., 2011; Malik et al., 2011; Teerlink et al., 2011, 2016) and decreases the proportion of myosin heads in the SRX conformation. Based on that, a recent study has proven OM to have a positive therapeutic impact in patients with heart failure and reduced ejection fraction (Teerlink et al., 2020). N-terminally truncated ELC has been shown to lead to stabilization of myosin SRX (Sitbon et al., 2020). Constitutive cRLC phosphorylation in the MYL2 R58Q variant has been shown to increase DRX myosin (Yadav et al., 2019a).

To better understand the role of myosin SRX in cardiac health and disease it is important to have a clear picture of its role within the sarcomere. Myosin SRX is primarily characterized by a decreased ATPase activity, which is correlated to and likely enabled by a specific orientation of myosin heads in relation to the thick filament, where the blocked and free myosin heads bind each other (Fig. 3) (Naber et al., 2011). This observation has been consistent with previously described switched-off states of myosin heads in non-muscle Myosin II forming a characteristic asymmetric J-like conformation which reduces ATP turnover (Craig and Woodhead, 2006; Jung et al., 2008). In the thick filaments of the sarcomere these SRX heads are ordered as they are conformationally uniform due to the intra- and intermolecular interactions that allow the SRX to form, as previously observed in tarantula muscle (Alamo et al., 2016; Wendt et al., 1999). Here the actin-binding site of the blocked head attaches to the ATP converter region of the free head (Wendt et al., 2001). While doing so the interacting heads of the myosin dimer bend back and bind to the thick filament helical rod-like core (Fig. 2A) (Woodhead et al., 2005). During this asymmetric conformation the phosphorylatable serine of the RLC on the free head becomes more exposed and freely accessible to MLCK whereas the serine on the blocked head becomes less accessible (Alamo et al., 2008). The head­­-head binding of the myosin dimer is not the only interaction that stabilizes myosin SRX, anchoring interactions between two adjacent myosin dimers have also been observed. These intermolecular binding events are located between the ELC of the blocked head of one myosin dimer and the free head of an adjacent myosin dimer (Woodhead et al., 2005). These intermolecular interactions have been suggested to contribute to cooperative activation in skeletal muscle (McNamara et al., 2015), and cardiac muscle. Cardiac muscle cooperative activation seems to be modulated by RLC phosphorylation, where phosphorylation prompts alterations in myosin orientation in neighbouring unphosphorylated myosin heads (Kampourakis and Irving, 2015). Five structural intramolecular interactions in total constitute the IHM including the aforementioned head-head interactions (Fig. 2A) (Alamo et al., 2016). The intramolecular interactions are proposed to keep the IHM in a compact off-structure, while the intermolecular interactions are suggested to allow the uniform formation of helical tracks of myosin heads on the surface of the backbone (Alamo et al., 2016; Yang et al., 2020). IHM interactions must be adjustable to regulate and maintain sarcomeric contractility an especially important characteristic to adapt cardiac output in the heart. The IHM regulatory complex includes the ELC, which possess a Ca^2+^ binding site, and RLC (Rayment et al., 1993; Scarff et al., 2020), which has a single serine in human myocardium that is phosphorylatable by the cardiac myosin light chain kinase (Chan et al., 2008; Chang et al., 2015; Davis et al., 2001; Ding et al., 2010). In addition to Ca^2+^ binding and RLC phosphorylation, stretch activation and mechanosensing have likewise been suggested to trigger the disruption of IHM interactions and thus initiate activation of the thick filament (Padrón et al., 2020). There is still much to be learnt about the IHM and its structural states, interactions, and dynamic physiologically driven regulation as well as its exact role and accountability for the structural and biochemical changes observed in the myosin SRX. Understanding the interplay between its function and structure will allow a deeper insight into disease pathomechanisms.

## Regulation of myosin SRX by protein level phosphorylation, and other key regulators

**Fig. 4.**
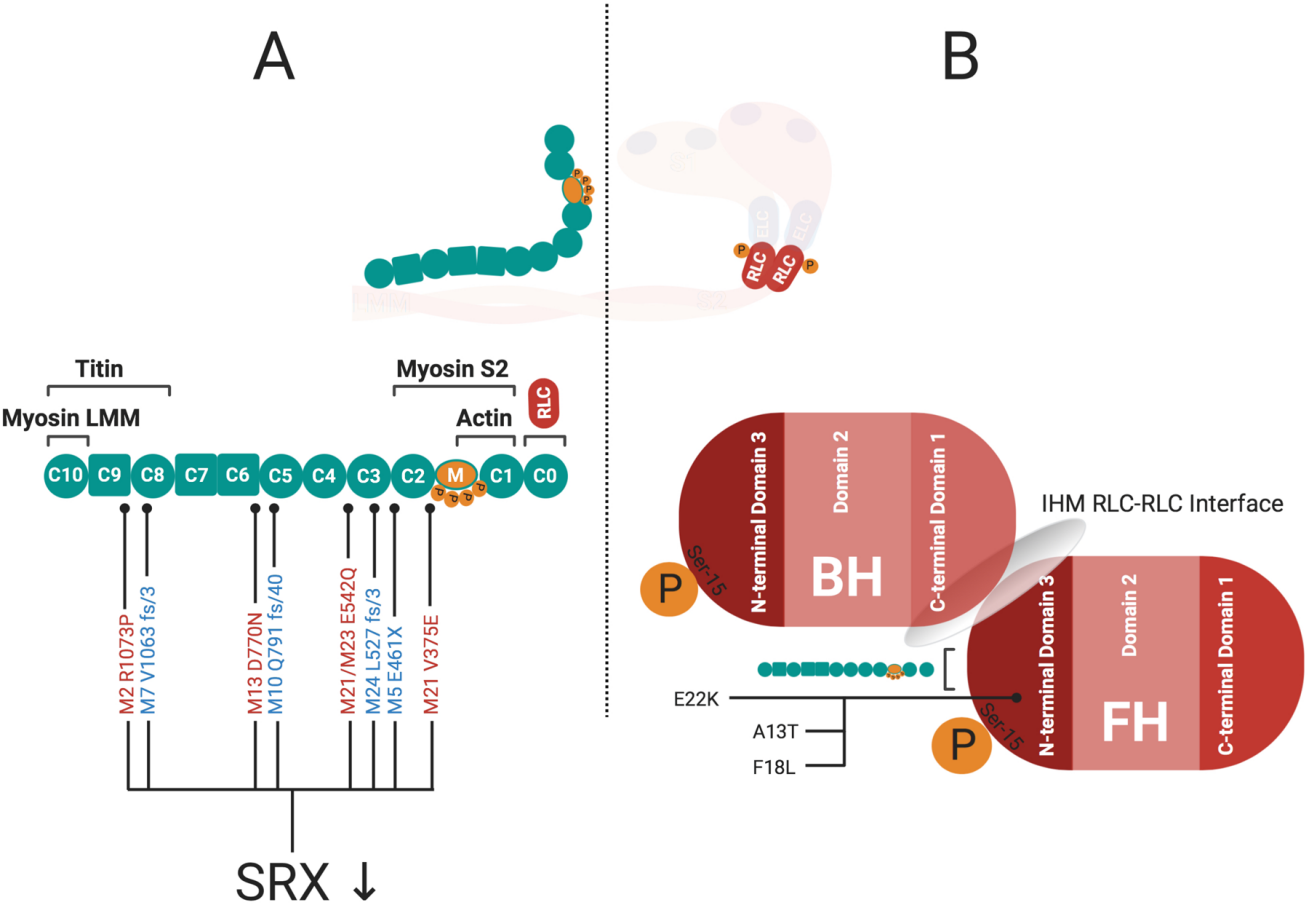
**The location and role of cMyBPC and cRLC mutations in HCM Pathogenicity.** (A) Truncating (blue) and missense (red) mutations in cMyBPC have been linked to HCM and also shown to reduce SRX proportions in the cardiac muscle (McNamara et al., 2017). It isn't clear how many of the missense variants in MYBPC3 could alter SRX. The cardiac specific CO domain of cMyBPC serves as a binding site for the N-terminal domain 3 of cRLC (Ratti, 2009). (B) The N-terminal domain 3 of cRLC itself contains the regulating phosphorylation site (Toepfer et al., 2013), functions as IHM RLC-RLC interface (Alamo et al., 2016), and has been shown to harbour HCM-related mutations (A13T, F18L, E22K) (Farman et al., 2014; Szczesna-Cordary et al., 2005), where the RLC E22K mutation in particular has been hypothesized to destabilize myosin SRX (Alamo et al., 2017).

The RLC and ELC at the neck region of myosin play an essential role in converting chemical energy into mechanical work as they are key players in altering the interactions between actin and myosin. RLC phosphorylation is a requirement for myosin function in smooth (Ikebe et al., 1994) and non-muscle myosin II, but acts as a modulator of contractile force and power in cardiac muscle (Heissler and Manstein, 2013; Toepfer et al., 2013). Phosphorylation of cardiac RLC (cRLC) occurs at serine 15 and is enacted primarily by the cardiac specific myosin light chain kinase (MLCK) (Scruggs et al., 2010). It has been demonstrated by EM and X-ray imaging, that the phosphorylation of RLC by MLCK in the striated muscle of the tarantula results in a loss of the helical ordered assembly of myosin heads, inferring a reduction in the organised myosin SRX (Fig. 3) (Padrón et al., 1991). Colson et al. (2010) have shown similar results by analysing the proximity of myosin cross bridge mass in relation to actin after treatment with cMLCK. cRLC phosphorylation lead to an increased abundance of cross-bridges in proximity to the thin filament reducing myosin SRX in cardiac muscle. The N-terminus of the RLC has shown to be essential to stabilize the IHM off state in smooth and skeletal muscle (Fig. 4) (Ikebe et al., 1994; Nogara et al., 2016). Until recently, it wasn't clear to what extent this N-terminal fragment was needed for regulation of SRX in cardiac muscle. Kampourakis et al. induced a 12 amino acid N-terminal truncation of the cRLC, which increased contractility much like phosphorylation of WT cRLC, increasing DRX myosin abundance (Kampourakis and Irving, 2015). This indicated that the N-terminus of cRLC in cardiac muscle is required to regulate SRX by stabilizing the parallel state of myosin heads with respect to the thick filament axis. Increasing sarcomere length analogously leads to a greater proportion of DRX myosin heads in the perpendicular state to the thick filament axis (Fig. 3) (Kampourakis et al., 2016). cRLC phosphorylation and sarcomere length act additively to alter DRX abundance and neither can fully activate the thick filament alone (Kampourakis et al., 2016). These findings support the notion that a certain proportion of cross-bridges/myosin heads remain in the SRX conformation (Fig. 1C) (Hooijman et al., 2011).

Structural insights into the IHM and SRX are supported by functional measurements in isolated trabecular muscle, where it has been shown that cRLC phosphorylation increased the rate of force re-development after muscle release (Sevrieva et al., 2020 and Toepfer et al., 2016b). This result inferred that the mechanism of action of RLC phosphorylation is the increase in Huxley's N_a_ parameter, which is consistent with a reduction of SRX and increase in DRX myosin states (Craig et al., 1987; Nag et al., 2017; Toepfer et al., 2016b). This has recently been validated in a synthetic thick filament system (Gollapudi et al., 2020). In addition phosphorylation of cRLC leads to a higher power production and V_max_ in trabecular muscle (Toepfer et al., 2013), inferring that cRLC phosphorylation is central to adapting sarcomeric power, which is at least partly mediated by altering IHM interactions. Experiments using the *in-vitro* motility assay found that cRLC phosphorylation increased actin sliding velocities proving that this mechanism is also partially independent of filamentous myosin (Karabina et al., 2015). Taken together, these results indicate the importance of cRLC phosphorylation as a modulator of cardiac contractile performance and suggest that cRLC phosphorylation could be a crucial target in therapeutic interventions. It is becoming clearer that cRLC phosphorylation is responsible for functional and orientational changes to myosin characteristics, but we are yet to establish if cRLC phosphorylation is only responsible for altering myosin states, or if it also alters intrinsic myosin properties such as the power stroke force and kinetics within the filamentous myosin (Greenberg et al., 2014). What complicates matters somewhat with using cRLC phosphorylation as a therapeutic target is the governance of cRLC phosphorylation and de-phosphorylation within the myocardium (Chang et al., 2016), and if the cell will counteract a therapeutic intervention.

Cardiac myosin-binding protein C (cMyBPC) also functions as a modulator of cross-bridge formation and force production (Fig. 3) (McClellan et al., 2001). cMyBPC is structurally integrated in the sarcomeric architecture as it binds to actin and titin in addition to myosin (Fig. 4A) (Lee et al., 2015). The C-terminal part of the protein is bound to the thick filament as observed in intact frog sartorius muscle and rat trabecular preparations (Luther et al., 2008, 2011), while the N-terminus with its cardiac specific C0 domain (Gautel et al., 1995) is attached to both myosin (Gruen and Gautel, 1999) and actin (Barefield and Sadayappan, 2010; Shaffer et al., 2009; van Dijk et al., 2014). Knocking out MYBPC3 in mice showed that sarcomeres without cMyBPC had reduced IHM conformations, which is now rationalised as an inability to form myosin SRX, slowing relaxation (Zoghbi et al., 2008). The structure and function of cMyBPC within the sarcomere is altered by phosphorylation and is likely to be phosphorylation site specific (Previs et al., 2016; Wang et al., 2014). There are several kinases that are able to phosphorylate cMyBPC (Barefield and Sadayappan, 2010). PKA-mediated phosphorylation of cMyBPC has been shown to lead to an increased proximity of cross bridges to the thin filament, facilitating interactions between myosin and actin (Colson et al., 2008). More recent studies have evaluated the impact of phosphorylation of cMyBPC on the structural state of myosin by specifically substituting the three main phosphorylatable serines of cMyBPC (Ser273, Ser282, Ser302) with either alanine (non-pshosphorylatable reidue) or aspartic acid (constitutive phosphorylation). These experiments compared the phosphomimetic state of cMyBPC to the nonphosphorylated state. It was found that filaments with the phosphomimetic cMyBPC have myosins that appear more disordered, while dephosphorylated cMyBPC displayed highly ordered cross bridge arrays insinuating an increased proportion of SRX myosins (Kensler et al., 2017). These structural studies have been followed up with single-nucleotide turnover experiments in skinned ventricular preparations where it was found that phosphorylation of cMyBPC reduced SRX myosins, which resulted in increased force production (McNamara et al., 2019). Most notably phosphorylation of Ser282 was sufficient for diminishing SRX (McNamara et al., 2019). Disrupting binding between cMyBPC and myosin subfragment-2 irrespective of cMyBPC phosphorylation results in decreased stabilization of the SRX (McNamara et al., 2019; Toepfer et al., 2019).

During contraction and relaxation the myosin filament backbone itself appears in several regulatory states depending on the different domains of the myosin filament (Fig. 1C) (Brunello et al., 2020). Specific regions of the thick filament have different proportions of myosins in the SRX and DRX states. These regions of the thick filament are regulated and important to its tightly controlled function during contraction and relaxation (Nelson et al., 2020). It is clear that there are many different physiological adapters of myosin SRX and each specific IHM interaction. There is an evolving picture of the importance of these confirmations in terms of contractile output of the sarcomere and energetic demand within the cardiomyocyte. It is not yet clear how these physiologic adapters of SRX and IHM interactions are altered in a variety of human diseases ranging from inherited to acquired conditions such as HCM, DCM, atrial fibrillation, diabetic cardiomyopathy, myocardial infarction, HFpEF and HFrEF.

## The role of myosin SRX in human disease and its utility as a druggable target

The myosin SRX conformation is an intricately controlled state within the myocardium. The interactions that allow the formation of myosin SRX are highly conserved throughout evolution. This has two significances when thinking about human disease (i) dysregulation of the myosin IHM and SRX in either inherited or acquired cardiac conditions would be severely detrimental to maintaining contractile and energetic homeostasis within the cardiomyocyte, and (ii) as a highly conserved set of interactions within the IHM that are central to contractility, relaxation, and energy usage, modulating myosin SRX is an attractive therapeutic target. HCM is estimated to affect at least 1 in 500 individuals (Maron et al., 1995; Semsarian et al., 2015), and is primarily caused by mutations in genes that control and encode sarcomeric proteins (Seidman and Seidman, 2001). The predominance ∼50% of HCM mutations are found in the genes encoding human beta cardiac myosin (MYH7) and cMyBPC (MYBPC3). The majority of the remaining HCM variants are found in other sarcomeric proteins such as TNNT2, TNNI3, TPM, MYL2 and MYL3 (Konno et al., 2010). In the case of MYH7 many of the mutations that cause HCM are clustered on a highly conserved surface of the catalytic domain of the myosin head. This region has recently been coined the cardiac myosin mesa and is one of the important IHM interaction sites in the myosin head domain (Fig. 2B) (Spudich, 2015). The surface of the myosin mesa consists of positively charged domains that could act as binding sites for the negatively charged surfaces on binding partners such as cMyBPC. Based on this it has been hypothesized that HCM causing mutations of the myosin mesa could cause decreased binding affinity for cMyBPC leading to an increased availability of the myosin head for binding to actin and contraction (Spudich, 2015; Trivedi et al., 2018). Pathogenic HCM mutations on the myosin mesa of either the blocked or the free head destabilize major IHM inter- and intramolecular interactions that are also integral for forming SRX (Alamo et al., 2017; Robert-Paganin et al., 2018; Spudich, 2019). Many HCM mutants in the mesa show increased numbers of swaying myosin heads (Adhikari et al., 2019; Sarkar et al., 2020) leading to hypercontractility, impaired relaxation and energy deprivation in cellular models of disease (Toepfer et al., 2020), key clinical phenotypes of HCM (Spudich, 2019; Toepfer et al., 2020). The dysregulation of SRX has been identified as a major determinant of cellular HCM pathophysiology accounting for impaired cellular relaxation, hypercontractility, and metabolic drain within the cell (Toepfer et al., 2020). There are multiple inter- and intramolecular interactions formed by the IHM that lie outside of the mesa that are likely to affect myosin SRX (Alamo et al., 2017; Homburger et al., 2016), which include regions of the myosin tail and S2 that are needed for allowing the sequestration of myosin heads against the thick filament backbone (Fig. 2A) (Adhikari et al., 2019). Outside of the HCM mutations in myosin the predominance of HCM mutations are located in MYBPC3 (Fig. 4A) (van Dijk et al., 2009), another protein that has a central role in the regulation of myosin SRX under physiological conditions. Variants that cause truncations in MYBPC3 predominantly lead to cellular haploinsufficiency (Marston et al., 2009). The lack of cMyBPC in the sarcomere releases the restricting effect of cMyBPC on myosin heads (Moss et al., 2015; Saber et al., 2008; Stelzer et al., 2006) decreasing myosin SRX, increasing contractility and slowing cellular relaxation, both central hallmarks of clinical HCM (McNamara et al., 2016; Toepfer et al., 2019). Certain missense variants found in the MYBPC3 gene cause HCM and have also been identified drivers of SRX depletion (Fig. 4A) (McNamara et al., 2017). But the direct mechanism that causes this is unclear, especially as the missense variants are found throughout the length of cMyBPC, and do not only cluster around the phosphorylation residues or myosin IHM binding regions (Fig. 4A).

As genetic sequencing technologies have evolved there is an ever greater pool of variants discovered in MYH7 and MYBPC3, these more recently identified variants often have no previously identified genotype to phenotype relationship. Identifying a genotype to phenotype relation is important to informing future clinical screening and follow-up. The bottleneck in understanding if a genotype is pathogenic or benign is a new hurdle for the field, where previously genotyping had been key to identifying genetic HCM. It is becoming ever more important to understand pathomechanisms of disease and genotype-phenotype relations when trying to understand HCM, especially as treatments move closer to personalised medicine. Not all variants within a specific gene may cause disease by the same mechanism, and indeed not all HCM variants across the multitude of classical HCM genes are likely to function through the same mechanisms. It is likely that the overarching classification of HCM is in fact a distinct subset of different diseases that manifest as classical HCM phenotypes at the organ level. Many well-studied pathogenic thick filament associated proteins when mutated are likely to interfere with IHM interactions and destabilise myosin SRX as a driving mechanism. But it is still unclear if IHM destabilization is a driving mechanism in many of the variants of unknown pathogenicity. This includes MYH7 missense variants outside of classical IHM sites, and MYBPC3 missense variants outside of myosin interacting domains (Fig. 4A). This extends to missense variants in MYL2 and MYL3, where the R58Q mutation of cRLC has been shown to increase myosin SRX, whilst structural predictions infer that many cRLC mutations may decrease myosin SRX (Alamo et.al., 2017). Deletion of the N-terminus of the ELC has been shown to increase myosin SRX (Fig. 4B) (Sitbon et al., 2020; Yadav et al., 2019a,b). Many of these variants are in close proximity to important IHM forming motifs in the IHM RLC-RLC interface, and may additionally interfere with RLC phosphorylation (Fig. 4B). Further experimental clarification will be needed to determine which of the variants found in these genes alter myosin SRX. In addition to this it is unclear how a significant proportion ∼30% of genotype positive HCM cases with variants in TNNT2, TNNI3, TPM and other thin filament associated variants would destabilise the IHM directly, as they do not interfere with residues on myosin. Indeed, it is quite likely that we may see an increase in SRX conformations of myosin in these HCM variants as a compensatory mechanism to reduce inappropriate hypercontractility (Repetti et al., 2019a). There is evidence that this is occurring as cRLC phosphorylation is depleted in thin filament HCM variants (Tucholski et al., 2020). This is an important distinction, both thin and thick filament HCM is typified by a hypercontractile phenotype and poor cellular relaxation, but this simplistically similar phenotype is driven by different mechanisms. These separate mechanisms may explain some of the differences in outcomes between patients that harbour variants in thick versus thin filaments (Coppini et al., 2014). Targeting the myosin ATPase with the allosteric myosin ATPase inhibitor Mavacamten has been shown to increase myosin SRX conformations (Fig. 3) by acting on multiple stages of the myosin chemomechanical cycle (Kawas et al., 2017), and in doing so relieves many of the organ, cellular, energetic, biochemical and also clinically apparent hallmarks of HCM in thick filament models of HCM (Anderson et al., 2018; Green et al., 2016; Jacoby et al., 2018; Olivotto et al., 2020; Rohde et al., 2018; Toepfer et al., 2020, 2019). Mavacamten has also been shown to partially restore Ca^2+^-sensitive molecular and cellular changes of HCM found in the TNNT2 R92Q and the TNNI3 R145G variants (Sparrow et al., 2020). However, a complete resolution of the HCM phenotype was not achieved by Mavacamten in thin filament variants. This may be because the direct mechanism of disease pathogenicity is not addressed by the action of Mavacamten (Repetti et al., 2019a). Thin filament HCM variants may have some benefit from increasing myosin SRX, but a novel therapeutic targeting the thin filament may be needed for complete resolution of sequelae.

In thinking about clinical non-ischemic genetically linked dilated cardiomyopathy (DCM) a disease that afflicts ∼1 in 250 people (Hershberger et al., 2013), there is a diverse genetic and environmental architecture that plays a role in disease outcome (McNally and Mestroni, 2017). By definition the disease is diagnosed by a reduction in contractile function characterised by a reduced fractional shortening and ejection fraction (Mestroni et al., 1999). At the cellular level this is often observed as a reduction in contractile function. Targeting myosin SRX in this setting may have a beneficial effect (Mamidi et al., 2017). Reducing myosin SRX would increase the proportion of available heads to form strong cross-bridges, which could salvage systolic insufficiency in DCM. This could be achieved by the positive cardiac inotropic compound Omeamtiv Mecarbil (OM) (Fig. 3) (Cleland et al., 2011; Kampourakis et al., 2018; Liu et al., 2018; Planelles-Herrero et al., 2017; Swenson et al., 2017; Teerlink et al., 2011; Woody et al., 2018). However, much like HCM, DCM is likely to be comprised of a far more complex sub-set of distinct pathomechanisms, so one drug targeting one specific mechanism may not salvage phenotype in all DCM variants.

Many forms of acquired cardiovascular disease manifest with changes in sarcomeric function. Some of these may be driven by changes in myosin SRX created by compensating levels of cMyBPC and cRLC phosphorylation among other protein modifications within the myocardium. Surviving myocardium within the chronically infarcted heart has been shown to initially compensate and increase power production, which is concomitant with increasing cRLC phosphorylation (Toepfer et al., 2016a). But the time-course of this compensation is not fully understood, with multiple models of myocardial infarction (MI) showing varying degrees of protein phosphorylation level change over time (Avner et al., 2012; van der Velden et al., 2011; Walker et al., 2010). One could hypothesise that post-MI in the failing myocardium there may be some benefit to increasing contractile function of the myocardium by employing OM, but it is not clear if this would eventually have a deleterious effect on cellular function as increasing DRX myosins would increase cellular energy demand on a cell likely to be exhibiting mitochondrial dysfunction (Zhou and Tian, 2018). As we do not yet know the effect of MI on SRX regulation this would be a good place to begin investigating the possible utility of therapeutically altering SRX to compensate heart failure. There is a wide variety of cardiac conditions that are diseases of muscular contractile insufficiency that may be partially mediated by myosin states, these include myocardial stunning (Bolli, 1990), heart failure with preserved ejection fraction (HFpEF) (Hamdani et al., 2013), and heart failure with reduced ejection fraction (HFrEF). MYH7 variants in the mesa have also been shown to have a higher incidence of atrial fibrillation compared to variants outside of the mesa region (Lee et al., 2018a,b). Atrial fibrillation is also a condition that often manifests with changes in cardiac contractile function, and has yet to be explored for changes in myosin SRX. Atrial fibrillation is an important atrial disorder, and we also have little understanding of SRX regulation in the atria as all investigations have used ventricular tissue. Changes in SRX abundance may even be a process that occurs during aging in skeletal muscle (Phung et al., 2018), but this is yet to have been investigated in the heart.

Great strides have been made towards understanding the regulation of muscle contraction in the heart in recent years. The discovery of SRX has provided important insight into the complex regulatory mechanisms of cardiac muscle activity in health and disease, but there is yet much to be understood.
